# DNA methyltransferase 3B expression is associated with poor outcome of stage I testicular seminoma

**DOI:** 10.1111/j.1365-2559.2012.04174.x

**Published:** 2012-05

**Authors:** Eri Arai, Tohru Nakagawa, Saori Wakai-Ushijima, Hiroyuki Fujimoto, Yae Kanai

**Affiliations:** 1 Division of Molecular Pathology, National Cancer Center Research InstituteTokyo, Japan; 2 Department of Urology, National Cancer Center HospitalTokyo, Japan

**Keywords:** DNA methyltransferase 3B, prognostication, seminoma, testicular germ cell tumour, tumour relapse

## Abstract

**Aims:**

To examine in testicular seminomas the expression of DNA methyltransferase 3B (DNMT3B), which is known to be associated with early embryonic development and carcinogenesis, and to obtain a predictive marker for relapse of stage I seminomas.

**Methods and results:**

Immunohistochemical examination of DNMT3B was performed in 88 cases of seminoma, 35 (39.8%) of which showed widely scattered nuclear immunoreactivity for DNMT3B, and 53 (60.2%) of which were completely negative. The incidence of focal DNMT3B expression was higher in stage III seminomas (5/5, 100%) than in stage I (25/70, 35.7%) or stage II (5/13, 38.5%) seminomas (*P* = 0.011). In stage I seminomas there were no significant correlations between DNMT3B expression and tumour size, invasion of the rete testis, or lymphatic or vascular involvement. Six of 25 cases (24%) showing DNMT3B expression relapsed, whereas only 3/45 cases (6.7%) lacking such expression did so (*P* = 0.037). Patients with seminomas showing DNMT3B expression had a significantly lower relapse-free survival rate than patients whose tumours lacked this feature (*P* = 0.0464).

**Conclusions:**

Patients with seminomas showing focal DNMT3B expression are at increased risk of relapse, and should be followed up carefully.

## Introduction

Seminoma is the most common histological subtype of testicular germ cell tumour (TGCT). At initial presentation, approximately 70–80% of patients with seminoma are diagnosed at clinical TNM stage I, i.e. showing no evidence of metastasis. However, 15–20% of them relapse after orchiectomy. As such relapse may occur 5 years or more after orchiectomy,^1^ careful long-term follow-up is necessary. However, prolonged follow-up and frequent radiological examinations are burdensome and costly.

Although postoperative radiotherapy of the para-aortic area was the standard choice for several decades,^2^ long-term follow-up studies have demonstrated an association of radiotherapy with late complications (i.e. cardiovascular disease^3^ and secondary malignancy). 4,5 In recent years, although single-course chemotherapy has yielded survival data equivalent to those after radiotherapy,^6^ its associated complications have not yet been evaluated in a long-term follow-up study. To avoid unnecessary treatment or over-frequent surveillance, markers predicting relapse risk are needed.

DNA methylation alterations play an important role in human carcinogenesis.^7^ With respect to TGCTs, a difference in methylated genes between seminoma and non-seminomatous TGCT has been reported.^8^,^9^ In addition, TGCT originates from male germ cells, and has various histological subtypes resembling the differentiation of fertilized germ cells to embryos. DNA methyltransferase 3B (DNMT3B) is a key enzyme for *de-novo* DNA methylation during the dynamic DNA methylation reprogramming that occurs in early embryogenesis.^10^ These facts suggest the possible participation of DNMT3B in TGCT development. However, only an association between DNMT3B expression and drug sensitivity of cultured embryonal carcinoma cells^11^ has been reported to date, and no studies have examined the clinicopathological significance of DNMT3B expression in TGCT. In this study, we examined the expression of DNMT3B protein in seminomas and explored the possibility of using it for prognostication.

## Materials and methods

### Patients and Tissue Samples

Eighty-eight testicular seminomas obtained from patients who underwent radical orchiectomy at the National Cancer Center Hospital, Tokyo, Japan were analysed. The mean (±standard deviation) age of the patients was 38.8 ± 9.2 years (range 21–66 years). The pathological diagnosis of TGCT was performed by two pathologists (E.A. and Y.K.), on the basis of the World Health Organization classification.^12^

Initial staging was carried out with a combination of chest X-ray and computed tomography scans. In this cohort, none of the patients received any preoperative therapy for the seminoma. None of the patients with stage I seminomas had received any postoperative therapy until relapse was diagnosed, and they had been under surveillance with chest X-ray, computed tomography scans, and determination of serum tumour marker levels (α-fetoprotein and the β-subunit of human chorionic gonadotropin).

This study was approved by the Ethics Committee of the National Cancer Center, Tokyo, Japan.

### Antibody

**Figure 1:**
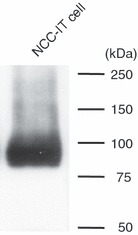
Western blotting with anti-human DNA methyltransferase 3B (DNMT3B) polyclonal antibody. A major immunoreactive band of about 95.8 kDa, corresponding to the molecular mass of DNMT3B, was detected in NCC-IT cells.

A polyclonal anti-human DNMT3B antibody was raised by immunizing rabbits with polypeptides corresponding to the C-terminal region of DNMT3B, N-ENKTRRRTADDSATS-C. To confirm the specificity of this antibody, NCC-IT cells, originating from human testicular teratoma,^13^ were subjected to western blotting analysis (Figure 1). Total cell extracts were separated by sodium dodecylsulphate (SDS) polyacrylamide gel electrophoresis and transferred electrophoretically to polyvinylidene difluoride (PVDF) filters, which were then incubated with the rabbit anti-human DNMT3B antibody (diluted 1:1000) overnight at 4°C, followed by horseradish peroxidase-linked anti-rabbit immunoglobulin (Amersham Biosciences, Buckinghamshire, UK). Finally, an ECL western blotting detection system (Amersham) was used for detection.

### Immunohistochemistry

Three-micrometre-thick sections of formalin-fixed, paraffin-embedded tissue specimens of each seminoma were deparaffinized and dehydrated. For antigen retrieval, the sections were heated for 10 min at 120°C in an autoclave. Non-specific reactions were blocked with 2% normal swine serum. All sections were incubated with the rabbit anti-human DNMT3B polyclonal antibody at 4°C overnight, and then with biotinylated secondary antibody (anti-rabbit IgG; dilution 1:200; Vector Laboratories, Burlingame, CA, USA;) at room temperature for 30 min. Following this, the sections were treated with Vectastain Elite ABC reagent (Vector Laboratories), and 3,3′-diaminobenzidine tetrahydrochloride was used as the chromogen. All sections were counterstained with haematoxylin. As a negative control, the primary antibody was omitted from the reaction sequence. Because high-level expression of DNMT3B mRNA has been reported in non-seminomatous TGCT, especially in embryonal carcinoma,^11^,^14^,^15^ we used three tissue specimens of testicular embryonal carcinoma as positive controls for immunohistochemistry.

### Statistics

Correlations between focal DNMT3B expression and clinicopathological features were analysed with the chi-squared test (*P* < 0.05). Survival curves of patients with and without focal DNMT3B expression in their testicular seminomas were calculated by the Kaplan–Meier method, and differences compared using the log-rank test.

## Results

### DNMT3B Expression in TGCT

**Figure 2:**
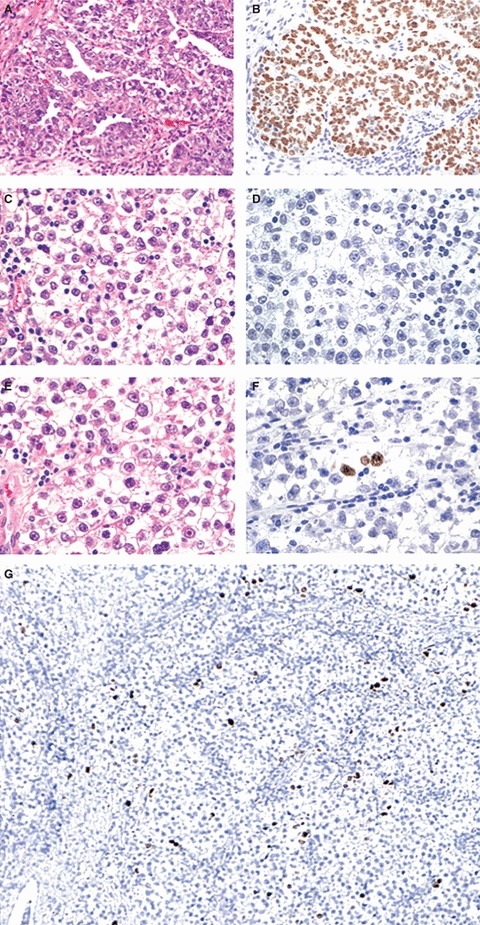
Haematoxylin and eosin staining (**A**,**C**,**E**) and immunohistochemistry with anti-human DNA methyltransferase 3B (DNMT3B) antibody **(B**,**D**,**F**,**G)** in testicular germ cell tumours. Embryonal carcinomas, as positive controls, showed diffuse and strong nuclear immunoreactivity for DNMT3B (**A**,**B**). Fifty-three of 88 seminomas were completely negative for DNMT3B (**C**,**D**), and scattered tumour cells with nuclear immunoreactivity were observed in the other 35 seminomas (**E**,**F**). DNMT3B-positive cells showed immunoreactivity as strong as that in embryonal carcinoma, whereas the surrounding tumour cells completely lacked immunoreactivity (**F**). There were no morphological differences between DNMT3B-positive and DNMT3B-negative cells (**E**,**F**). Widely scattered nuclear immunoreactivity for DNMT3B was seen at low magnification (**G**).

All three samples of embryonal carcinoma used as positive controls showed diffuse and strong immunoreactivity for DNMT3B (Figure 2A,B). Immunoreactivity was observed in the nuclei, but not in either the cytoplasm or the cell membrane.

In contrast to embryonal carcinomas, most seminomas were completely immunonegative for DNMT3B (Figures 2C,D). However, in some seminomas, tumour cells showing strong nuclear immunoreactivity were scattered among a large majority of tumour cells lacking nuclear immunoreactivity (Figure 2E,F,G).

On the basis of these findings, we considered a seminoma to be positive for (focal) DNMT3B expression when one or more tumour cells per 50 high-power fields showed strong nuclear immunoreactivity equivalent to that of embryonal carcinoma. With the use of this criterion, 35/88 seminomas (39.8%) were considered to be positive. No seminomas showed diffuse immunoreactivity for DNMT3B.

There were no significant morphological differences between the scattered tumour cells showing immunoreactivity and the surrounding tumour cells lacking immunoreactivity in each individual seminoma (Figure 2E,F). Furthermore, there were no significant histological differences between seminomas with focal DNMT3B expression and those lacking it.

### Correlation Between Focal DNMT3B Expression and Clinicopathological Parameters of Patients with Seminomas

Table 1 shows the correlation between focal DNMT3B expression and initial tumour stage in patients with seminomas. The incidence of DNMT3B expression was significantly higher in stage III seminomas (100%) than in stage I (35.7%) or stage II (38.5%) seminomas (*P* = 0.011), suggesting that such expression may reflect tumour aggressiveness. Therefore, we analysed the correlation between DNMT3B expression and various clinicopathological parameters in stage I seminomas. There were no significant correlations between expression and tumour size, invasion of the rete testis, invasion of the epididymis, lymphatic or vascular involvement, or invasion of the spermatic cord (Table 2). On the other hand, 9/70 patients with stage I seminoma suffered tumour relapse during follow-up after orchiectomy (metastasis to the retroperitoneal lymph nodes in eight and to the lumbar vertebra and humerus in one). Six of 25 cases (24%) showing DNMT3B expression developed distant metastases, whereas only 3/45 cases (6.7%) lacking expression did so (*P* = 0.037; Table 2).

**Table 1:** Correlation between focal DNA methyltransferase 3B (DNMT 3B) expression and initial TNM stage in patients with seminomas

	Focal DNMT3B expression		
		
Initial TNM stage	Negative	Positive	P*
Stage I	45	25	0.011
	
Stage II	8	5	
	
Stage III	0	5	

* Chi-squared test.

**Table 2:** Correlations between focal DNA methyltransferase 3B (DNMT3B) expression and clinicopathological parameters in stage I seminomas

	Focal DNMT3B expression		
		
Clinicopathological parameters	Negative	Positive	P*
Tumour size (mm)			
≤40	16	8	0.764
	
>40	29	17	

Invasion of the rete testis†			
Negative	13	10	0.343
	
Positive	32	15	

Invasion of the epididymis			
Negative	44	24	0.669
	
Positive	1	1	

Vascular involvement			
Negative	36	15	0.071
	
Positive	9	10	

Invasion of the spermatic cord			
Negative	44	23	0.253
	
Positive	1	2	

Tumour relapse			
Negative	42	19	**0.037**
	
Positive	3	6	

* Chi-squared test.

† All positive cases showed interstitial invasion but not Pagetoid spread.

The *P*-value of <0.05, which indicates significant differences, is in bold.

### Prognostic Significance of Focal DNMT3B Expression in Stage I Seminoma

**Figure 3:**
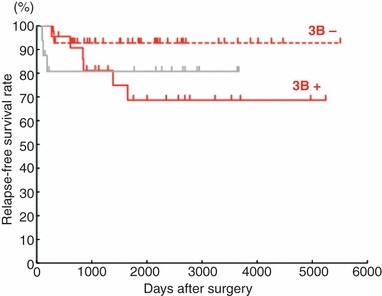
Kaplan–Meier survival curves of patients with stage I testicular germ cell tumours (TGCTs). The relapse-free survival rate of patients with seminomas showing focal DNMT3B expression (red solid line) was significantly lower than that of patients with seminomas not showing this feature (red broken line, *P* = 0.0464). There was no significant difference between the relapse-free survival rate of patients with seminomas showing focal DNMT3B expression and that of patients with non-seminomatous TGCTs (gray line, *P* = 0.747). The time to relapse of patients with seminomas showing DNMT3B expression tended to be longer than that of patients with non-seminomatous TGCTs.

Figure 3 shows the Kaplan–Meier survival curves of patients with stage I seminomas. The period covered ranged from 15 to 5509 days (mean 1794 days). The relapse-free survival rate of patients with seminomas showing DNMT3B expression was significantly lower than that of patients with seminomas lacking expression (*P* = 0.0464).

## Discussion

Postoperative management of stage I seminoma has been the subject of active discussion among clinicians and researchers.^16^–^18^ Although both postoperative radiotherapy and chemotherapy can reduce the rate of relapse in patients with stage I seminomas,^6^ patients who do not suffer relapse may be overtreated. As curative chemotherapy is available for metastatic seminoma if it can be detected early, careful surveillance is thought to be an effective strategy for avoiding overtreatment.^17^ Estimation of relapse risk may be advantageous for appropriate surveillance planning in individual patients with stage I seminoma.

Several groups have studied prognostic factors in stage I seminoma. On the basis of a pooled analysis, Warde *et al.* reported that tumour size (≤40 mm or >40 mm), invasion of the rete testis and lymphatic or vascular involvement were predictive of relapse on univariate analysis, and that tumour size and invasion of the rete testis remained significant on multivariate analysis.^19^,^20^ However, other papers did not report these factors to be significantly predictive. In our cohort, although lymphatic or vascular involvement was significantly correlated with tumour relapse (*P* = 0.0304), tumour size and invasion of the rete testis were not (*P* = 0.1445 and *P* = 0.0566, respectively, log-rank test). Thus, any prognostic impacts of these clinicopathological parameters are still controversial. Moreover, no significant molecular marker for prognostication of patients with seminoma has yet been identified.

In the present study, we found, using immunohistochemistry, that seminomas were separable into two groups on the basis of DNMT3B protein expression: focal DNMT3B expression with widely scattered nuclear reactivity, and absence of such expression. Patients with seminomas showing focal DNMT3B expression were frequently diagnosed at a higher stage (Table 1), and had a poorer outcome (Figure 3), than patients with seminomas lacking such expression.

Tickoo *et al.*^21^ suggested that seminomas with ‘atypical features’, based on morphology, positive expression of CAM5.2 and/or CD30, and negative expression of CD117, tended to be more aggressive, with a higher initial tumour stage. When our cohort of seminomas was examined on the basis of the same methods and criteria,^21^ CAM5.2 reactivity (15/87 cases, 17.2%) was significantly correlated with widely scattered nuclear reactivity for DNMT3B (*P* = 0.0028), whereas histological atypia (*P* = 0.321) and expression of CD30 (*P* = 0.0653) and CD117 (*P* = 0.0814) did not show such significant correlations. CAM5.2 reactivity did not correlate significantly with any of the clinicopathological parameters in Table 2, including tumour relapse (*P* = 0.949) or relapse-free survival rate (*P* = 0.6300, log-rank test).

The relapse-free survival rate of patients with seminomas showing focal DNMT3B expression was as low as that of the 16 examined patients with stage I non-seminomatous TGCTs who underwent orchiectomy at the NCCH, Tokyo, Japan (*P* = 0.747; Figure 3), although previously patients with seminomas have been generally considered to have a more favourable outcome than those with non-seminomatous TGCTs. 22,23 Moreover, seminomas showing DNMT3B expression tended to relapse later than non-seminomatous TGCTs (Figure 3). These data indicate that DNMT3B expression may be a potentially useful indicator for estimation of relapse risk in patients with stage I seminoma: patients whose tumours show such expression should be followed up as closely as, and for a longer period than, patients with non-seminomatous TGCTs.

Immunohistochemistry may be the only method capable of detecting widely scattered nuclear reactivity: analyses of mRNA and protein in tissue and serum samples using other methods may not be able to demonstrate DNMT3B expression in only a small number of scattered tumour cells. Thus, immunohistochemistry for DNMT3B, which can be performed on the formalin-fixed, paraffin-embedded tissue specimens prepared for routine pathological diagnosis, may be clinically useful for prognostication of stage I seminomas after prospective validation.

According to the histogenetic model proposed by Srigley *et al.*,^24^ seminoma has the potential to differentiate to embryonal carcinoma or other non-seminomatous TGCTs, resembling the differentiation of fertilized germ cells to embryos. In fact, in patients with testicular seminomas who suffer relapse, the metastatic lesions show histological subtypes of embryonal carcinomas and other non-seminomatous TGCTs. *De-novo* DNMT, Dnmt3b, contributes to dynamic epigenetic reprogramming, especially in early mammalian embryogenesis,^10^ and is expressed in pluripotent cells, such as the inner cell mass and embryonic ectoderm cells of mouse embryos, and in isolated embryonic stem cells.^25^ Embryonal carcinomas also showed high-level expression of DNMT3B (Figure 2A,B).^11^,^14^,^15^ DNMT3B may be associated with cell pluripotency through its *de-novo* DNA methylation ability, not only during development but also in TGCTs. Focal DNMT3B expression may reflect the potential for differentiation to embryonal carcinomas and other non-seminomatous TGCTs in a proportion of tumour cells in stage I seminoma. It is feasible that seminomas with such potential would relapse as frequently as non-seminomatous TGCTs after a period long enough to allow such differentiation to occur.

## Abbreviations

DNMT: DNA methyltransferase
TGCT: testicular germ cell tumour
